# Rapid Determination of Active Compounds and Antioxidant Activity of Okra Seeds Using Fourier Transform Near Infrared (FT-NIR) Spectroscopy

**DOI:** 10.3390/molecules23030550

**Published:** 2018-03-02

**Authors:** Fangbo Xia, Chenchen Li, Ning Zhao, He Li, Qi Chang, Xinmin Liu, Yonghong Liao, Ruile Pan

**Affiliations:** Institute of Medicinal Plant Development, Chinese Academy of Medical Science, Peking Union Medical College, Beijing 100193, China; xiafb08@163.com (F.X.); nutritionality@163.com (C.L.); ningzhao7@163.com (N.Z.); li_he@163.com (H.L.); qchang@implad.ac.cn (Q.C.); liuxinmin@hotmail.com (X.L.); yhliao@implad.ac.cn (Y.L.)

**Keywords:** FT-NIR, polyphenols, flavonoids, antioxidant activity

## Abstract

Okra seeds (OSD) have been proved to possess significantly anti-fatigue activity and due to their high contents of flavonoids and polyphenols. While, the quality of OSD is easily affected by harvest time, region and other factors. In this research, the rapid method based on Fourier transform near infrared (FT-NIR) spectroscopy was developed for quality assessment of okra seeds. Firstly, 120 samples’ spectra were acquired, and quantification of isoquercitrin, quercetin-3-*O*-gentiobioside, total phenols (TP) and antioxidant assays including 1-diphenyl-2-picrylhydrazyl (DPPH) scavenging, ferric reducing antioxidant power (FRAP) were conducted. Next, partial least squares (PLS) regression and full cross-validation were applied to develop calibration models for these data, and external validation was used to determine models’ quality. The coefficient of determination for calibration ($R_{c}^{2}$), the root mean square error of cross validation (RMSECV) and the corresponding determination coefficients for cross-validation ($R_{\mathrm{cv}}^{2}$) proved all these models have excellent precision. Besides, the residual predictive deviation (RPD) of models (4.07 for isoquercitrin, 4.04 for quercetin-3-*O*-gentiobioside, 9.79 for TP, 4.58 for DPPH and 4.12 for FRAP) also demonstrated that these models possessed good predicative ability. All these results showed that FT-NIR spectroscopy could be used to rapidly determine active compounds and antioxidant activity of okra seeds.

## 1. Introduction

Okra (*Abelmoschus esculentus* (L.) Moench, Malvaceae Family), also known as lady’s finger, bhindi and gumbo, is an annual plant native to Africa and has been grown in different countries around the world, mainly in tropical, subtropical and warm temperate regions [1]. The pods of okra have long been used as a vegetable and a source of dietary medicine [2]. Previous studies have reported that okra seeds contained high contents of polyphenols and flavonoids [3,4], which have been proved possess significant antioxidant [3,5,6], anti-fatigue [7], anticancer [8] and other activities. Therefore, it is promising to develop okra seeds into functional foods. While, our preliminary researches have showed the contents of flavonoids and polyphenols in okra seeds were easily affected by harvest time, planting region and other factors. Therefore, it is essential to build rapid and robust analytical methods to evaluate quality of okra seeds by analyzing the contents of flavonoids, polyphenols and the antioxidant activity.

Currently, chemical methods including Folin–Ciocaiteu method [9,10], colorimetric aluminum chloride method [11,12] and high performance liquid chromatography (HPLC) [13,14] were widely used to determine the contents of polyphenols and flavonoids in medicinal plants. Meanwhile, 1-diphenyl-2-picrylhydrazyl (DPPH) scavenging, ferric reducing antioxidant power (FRAP), oxygen radical absorption capacity (ORAC) were usually applied for testing antioxidant activity [7,15,16,17]. Although these methods can provide reliable, accurate description of okra seeds quality, they are destructive, expensive and time-consuming sample. 

Nowadays, Fourier transform near infrared (FT-NIR) spectroscopy has been widely applied to determine the quality of various foods and herbs [18,19,20]. NIR is the part of the electromagnetic spectrum, lies between the visible and IR regions of the electromagnetic spectrum, and is usually defined by the wavelength range 14,000–4000 cm^−1^ [21,22]. It provides much more complex structural information related to the variation behaviors of combinations of bonds [22]. The technique has been used to predict different groups of compounds, including volatile compounds (esters, higher alcohols and fatty acids) in apple wines [21], polyphenols in red grape skins and green tea [23,24]. Meanwhile, it is also reported to rapidly detect the antioxidant activity of bamboo leaf extract and moravian wines [25,26].

Compared with chemical methods mentioned above, NIR technology is fast, non-destructive, efficient and can detect several parameters simultaneously. However, no reports were found in relation to the use of NIR spectroscopy in the monitoring and determination of antioxidant constituents and activity of okra seeds. The aim of this research was to apply spectroscopic technology for analysis of antioxidant compounds and activity of okra seeds.

## 2. Results and Discussion

### 2.1. Chemical Determination and Antioxidant Activity Assays

In this research, 120 samples were determined for reference data with classic methods including the contents of isoquercitrin, quercetin-3-*O*-gentiobioside, total polyphenols (TP), and assays of DPPH and FRAP. As shown in the Table 1, the contents of isoquercitrin, quercetin-3-*O*-gentiobioside and TP showed the similar range for calibration and validation sets. Besides, standard deviation (SD) and standard error (measurement of uncertainty) of laboratory method (SEL) of isoquercitrin, quercetin-3-*O*-gentiobioside, TP also showed no significant difference. 

Due to the complex of antioxidant compounds in okra seeds, the contents of TP and flavonoids cannot completely reflect the antioxidant activity. DPPH and FRAP were used to measure the antioxidant activities. As seen from Table 1, for antioxidant activities, the scope of reference results in the calibration set roughly covers the scope in the prediction set, and their SD and SEL values between the two sets are no significant differences. Therefore, the division of samples is appropriate.

### 2.2. Calibration Models Development

**Figure 1:**
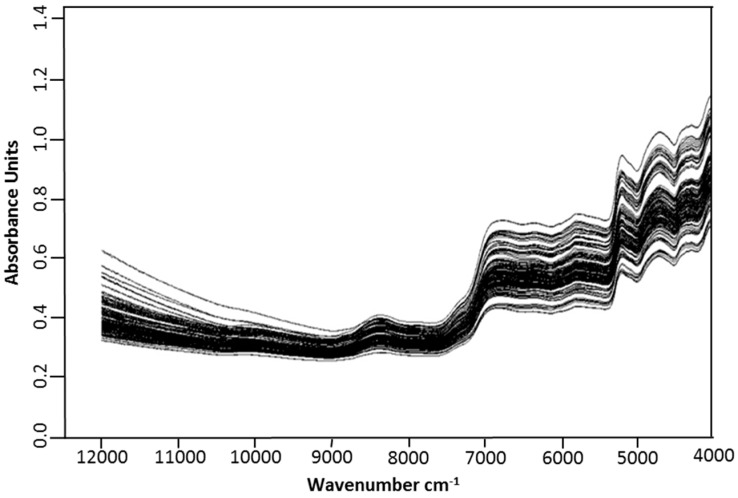
Raw Fourier transform near infrared (FT-NIR) spectra of researched samples.

Since the obtained spectral data (Figure 1) contained not only sample information, but also background information and noises, it is important to preprocess spectral data before model building [27]. As shown in Table 2 and Supplementary materials (Figures S1–S3), it could be concluded that the processed NIR spectra with FD (first derivative) + SNV (stand normal variate) methods have the best ability for determination of isoquercitrin (effective wavenumber, 11,995.5~6098; 4601.5~4246.1 cm^−1^), quercetin-3-*O*-gentiobioside (effective wavenumber range, 7502~5446.2; 4601.5~4246.6 cm^−1^) and TP (effective wavenumber range, 7502~5446.2; 4601.5~4246.6 cm^−1^) compared to other models due to the highest R^2^ value, and the lowest RMSEC, RMSECV and RMSEP, but minimum difference between RMSECV and RMSEP [21]. Besides, the RPD value higher than three is recommended for screening purposes, and the RMSECV values of the model was lower than 2 (RMSEC) and higher than RMSEC as well as the values of $R_{\mathrm{cv}}^{2}$ higher than 0.90 indicate the model have excellent precision [21]. Similarly, it also could be seen that the preprocessed data by MSC method were proved to be most appropriate to build models for DPPH (effective wavenumber range, 11,995.5~4246.6 cm^−1^) and FRAP (effective wavenumber range, 7502~4246.5 cm^−1^) in Table 2 and Supplementary materials (Figures S4 and S5).

As for the comparison between RMSEC and SEL, for the Table 1 and Table 2, it could be seen that SEL values for each parameter (isoquercitrin, quercetin-3-*O*-gentiobioside, TP, DPPH and FRAP) were lower than RMSEC values, which demonstrated that the accuracy of FT-NIR methods built in this research were lower than standard methods. 

### 2.3. External Validation

**Figure 2:**
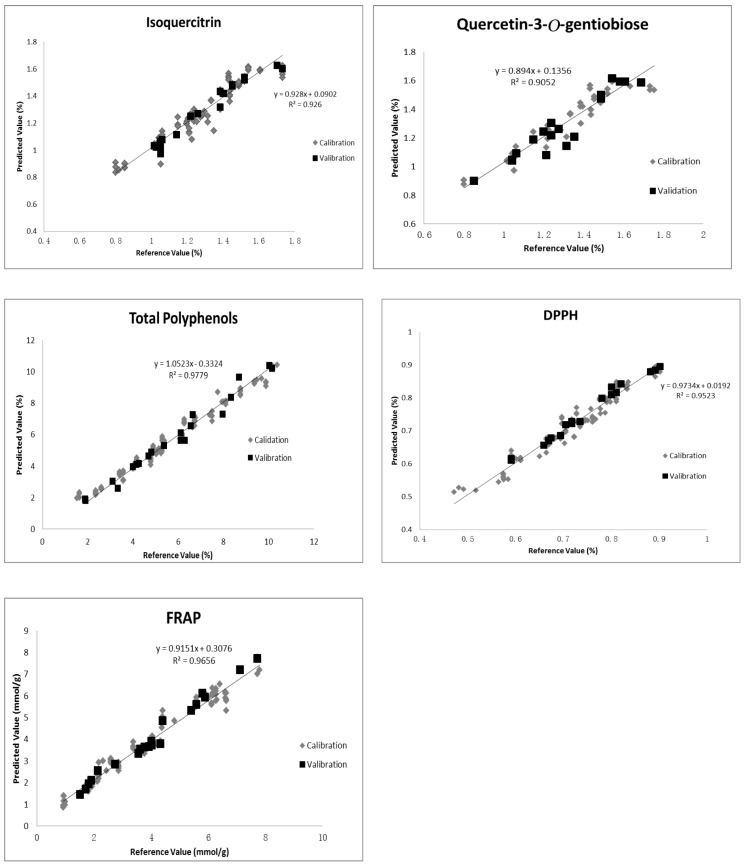
Correlation between reference values and FT-NIR predicted values using PLS for okra seeds. DPPH: 1-diphenyl-2-picrylhydrazyl; FRAP: Ferric Reducing Antioxidant Power.

To inspect the predictive ability of models, the external validation procedure was conducted in this research. Spectra of 20 okra seed samples, which were not included in the calibration set, were obtained for three times, and the spectral average was taken. Subsequently, the selected models for isoquercitrin, quercetin-3-*O*-gentiobioside, TF, DPPH and FRAP were used to predict the values, and then compared the predicted values with measured values. As shown in Table 3, the statistics of determination for validation ($R_{p}^{2}$) were all higher than 0.90, and even higher than 0.95 for TP, DPPH and FRAP, and the RPD values of isoquercitrin, quercetin-3-*O*-gentiobioside, TP, DPPH and FRAP were higher than 3. Besides, measured values versus FT-NIR predicted values from the optimal models were plotted in Figure 2, and regression equations and regression coefficients were also presented in Figure 2. These data indicated PLS models for isoquercitrin, quercetin-3-*O*-gentiobioside, TP, DPPH and FRAP possessed excellent predictive ability.

## 3. Materials and Methods

### 3.1. Chemicals and Samples

Isoquercitrin, quercetin-3-*O*-gentiobioside and gallic acid standards were purchased from National Institute for Food and Drug Control (Beijing, China). Folin-Ciocalteu’s phenol reagent (1N) was purchased from Coolaber Science & Technology (Beijing, China). Ferric reducing antioxidant power (FRAP) kit was purchased from Beyotime Biotechnology (Beijing, China). 1,1-diphenyl-2-picrylhydrazyl (DPPH) and trolox were purchased from Sigma-Aldrich Inc. (St. Louis, MO, USA). Phosphate buffer (PBS, Gibco) was purchased from Thermo Fisher (Waltham, MA, USA). The other reagents used in the research were of analytical grade. 

Fresh okra seed samples were collected from major production areas of China, and three batches (*n* = 40 per batch) were randomly selected for every region from May to September in 2015. For every batch, 2000 g fresh okra were randomly collected and their seeds were dried at 60 °C with a DHG-9140A drying oven (Yiheng Instruments, Shanghai, China) for 8 h. There were 120 samples in total and every dried sample was pulverized with BJ-800A drug pulverizer (Baijie) and sieved through a 20 mesh sieve to yield a fine powder. All sample powders were stored a desiccator for further study. 

For the determination of reference data, 1 g of okra seed powder was extracted with 20 mL deionized water at 100 ± 1 °C for 1 h (3 times) and filtered thereafter. The extract thus obtained was concentrated by removing the water under vacuum and freeze drying. All extractive samples were stored in a desiccator at room temperature for further study.

### 3.2. The Determination Of Active Compounds

#### 3.2.1. Determination of Isoquercitrin and Quercetin-3-*O*-gentiobioside Contents

Quantification of isoquercitrin and quercetin-3-*O*-gentiobioside was performed by HPLC-UV using a five-point calibration curve (r^2^ = 0.999) in the range of 5–500 μg/mL [7]. Isoquercitrin and quercetin-3-*O*-gentiobioside (5 mg, respectively) and samples (10 mg) were dissolved in 10 mL methanol, respectively. All solutions were filtered through 0.45 μm polytetrafluoroethylene filters before HPLC analysis. The Waters 600 HPLC pump combined a Waters 2070 autosampler and a Waters 2489 UV/Visible detector were employed to analyse chemical compounds through a reversed phase column (Thermo BDS HYPERSIL C18, 4.6 mm × 250 mm, 3 μm) at a flow rate of 1 mL/min. The mobile phase consisted of acetonitrile (28%) and 0.1% acetic acid in water (72%). The wavelength for UV detection was 354 nm and the column temperature was set at 25 °C. The compounds were identified by comparing with the retention time of standards, and quantified through calculating the area under the curve with external standards.

#### 3.2.2. Determination of TP Content

The TP content was determined using Folin-Ciocaiteu method [7]. In short, 50 mg of extract for each sample was mixed with 25 mL of 50% methanol solution, then 0.5 mL sample solution, 0.3 mL Folin–Ciocaiteu’s reagent and 10 mL sodium carbonate (10%) were sufficiently mixed, and then the volume was adjusted to 25 mL with distilled water. The mixture was allowed to stand at 50 °C in darkness for 1 h. Absorbance was measured at 765 nm. A calibration curve of Gallic acid was prepared. The results were expressed as mg of Gallic acid equivalents per 100 mg of dried okra seeds.

### 3.3. Antioxidant Activity Measurement

The antioxidant capacity of each sample were detected by 1-diphenyl-2-picrylhydrazyl (DPPH) scavenging and ferric reducing antioxidant power (FRAP) with slight modification [7], and trolox was used as the positive control. Results were expressed as trolox equivalent antioxidant capacity.

#### 3.3.1. DPPH Radical Scavenging Activity

For DPPH assay, fifty microliters sample solution (0.4 mg okra seeds powder/mL) was mixed with DPPH solution (100 μL, 1.28 × 10^−4^ mol/L) for measurement of free radical-scavenging activity (A_1_) and 95% ethanol (100 μL) for the control (A_2_). Distilled water (50 μL) was mixed with DPPH solution (100 μL) for the blank (A_0_). The absorbance was measured at 517 nm after the solutions were mixed and kept at room temperature for 30 min. The capacity to scavenge DPPH radical was calculated using the following equation:

Scavenging activity (%) = [1 − (A_1_ − A_2_)/A_0_] × 100%. (1)

#### 3.3.2. Ferric Reducing Antioxidant Power (FRAP)

The FRAP assays of all samples were detected according to instruction of Beyotime Institute of Biotechnology. For each sample, the diluted sample solution (5 μL, 0.4 mg dried okra seeds/mL) was mixed with FRAP working solution (180 μL) and kept for 5 min at 37 °C. The absorbance of the reaction mixture was then recorded at 593 nm. The standard curve was prepared using FeSO_4_, ranging from 0.15 to 1.5 mM.

### 3.4. Spectral Acquisition

The NIR spectra were obtained in the diffuse reflectance mode using the MPA multi-purpose FT-NIR analyzer (Bruke, Karlsruhe, Germany) equipped with a quartz beam splitter; an integrated Michelson interferometer; highly sensitive PbS detector, multiple NIR measurement accessories for different sampling techniques combined with Opus 6.0 software (Bruke, Karlsruhe, Germany). In this study, all samples’ spectra were collected in diffuse reflectance mode with sphere macrosample integrating sphere measurement channel and a gold background was used for the reference. For each sample, fifty grams of okra seed powder was densely packed into a sample cup and placed in sample rotator for a high reproducibility and avoiding any inhomogeneity in sample. 

The spectra generated over a range of wave numbers from 12,000 cm^−1^ to 4000 cm^−1^. The scanner speed was 10 kHz and each spectrum was the average of 64 scanning spectra. And for each sample, three reflectance spectra were obtained and the average of them was used as the data of this sample in calibration or prediction.

### 3.5. Data Analysis

Random allocation was applied to divide samples (*n* = 120) into calibration (*n* = 100) and validation (*n* = 20) sets. Instrument control, collection of spectrum data and chemometric analysis were performed using OPUS software (v.5.5 Bruker Optics, Ettlingen, Germany). In order to improve spectral features and to further build a robust prediction model, various techniques including first derivative (FD), second derivative (SD), multiplicative scatter correction (MSC), min/max normalization (MMN), stand normal variate (SNV), minus a straight line (MSL), constant offset elimination (COE), straight line subtraction (SLS) and others, were screened in this research. The OPUS software package (Bruke, Germany) was used for processing the data, and then processed spectral data were analyzed with PLS calibration techniques. Models were formulated which related the FT-NIR spectra and the reference chemical values (isoquercitrin, quercetin-3-*O*-gentiobiose, TP, DPPH and FRAP) in each sample of okra seed. Statistically, PLS method can simplify the correlation between X-data (spectral data) and Y-data (reference chemical data) by assuring that all latent variables are arranged on the basis of their relevance for predicting Y. In this research, a cross validation method was applied in model validation with as several validation subsets as there were samples involved in the calibration model (leave-one-out method). 

The accuracy of the calibration models is evaluated with the coefficient of determination for calibration set ($R_{C}^{2}$) and the root mean square errors assessed by cross-validation (RMSECV), which were calculated according to Equations (2) and (3):

(2) $$R_{C}^{2}=1-\frac{\sum_{i=1}^{n}{\left({\hat{y}}_{\mathrm{ci}}-y_{\mathrm{ci}}\right)}^{2}}{\sum_{i=1}^{n}{\left({\hat{y}}_{\mathrm{ci}}-y_{\mathrm{ci}}\right)}^{2}}$$

(3) $$\mathrm{RMSECV}=\sqrt{\frac{\sum_{i=1}^{n_{c}}{\left({\hat{y}}_{\mathrm{ci}}-y_{\mathrm{ci}}\right)}^{2}}{n_{c}}}$$

where n_c_ is the number of samples in the calibration set, $y_{\mathrm{ci}\;}$ is the reference measurement value obtained from chemical methods for the sample i, ${\hat{y}}_{\mathrm{ci}}$ is the predicted value by NIR spectra for sample i, and ${\bar{y}}_{\mathrm{ci}}$ is the mean of the reference measurement results for all samples in the calibration set.

The prediction accuracy of the calibration model was tested by the coefficient of determination for the prediction set ($R_{\mathrm{CV}}^{2}$) and root mean square error of prediction (RMSEP), which were calculated by Equations (4) and (5):

(4) $$R_{\mathrm{CV}}^{2}=1-\frac{\sum_{i=1}^{n}{\left({\hat{y}}_{\mathrm{ci}}-y_{\mathrm{pi}}\right)}^{2}}{\sum_{i=1}^{n}{\left({\hat{y}}_{\mathrm{ci}}-{\bar{y}}_{\mathrm{pi}}\right)}^{2}}$$

(5) $$\mathrm{RMSEP}=\sqrt{\frac{\sum_{i=1}^{n_{p}}{\left({\hat{y}}_{\mathrm{pi}}-y_{\mathrm{pi}}\right)}^{2}}{n_{p}}}$$

where n_p_ is the number of samples in the prediction set, $y_{\mathrm{pi}}$ is the reference measurement value obtained from chemical methods for the sample i, ${\hat{y}}_{\mathrm{pi}}$ is the predicted value by NIR spectra for sample i by the model developed when the *i*th sample is left out, and ${\bar{y}}_{\mathrm{pi}}$ is the mean of the reference measurement results for all samples in the prediction set.

Apart from these parameters, the value of residual predicative derivation (RPD), which was calculated by Equations (6), was used to standardize the predictive accuracy in this study:

(6) $$\mathrm{RPD}=\frac{S.D.}{\mathrm{RMSEP}}$$

where S.D. is the standard deviation for the prediction samples. RPD value is used to check robustness of a model, and relatively higher RPD value indicates a better capability for prediction. Generally, a cut-off point of 3 is recommended by researchers, and it is widely accepted that the model with a higher RPD value has a good prediction performance. 

## 4. Conclusions

This research has proved that FT-NIR spectroscopy can be applied to rapidly determine the contents of isoquercitrin, quercetin-3-*O*-gentiobioside, total polyphenols and antioxidant activity of okra seeds. Moreover, these models developed in this study possess a good ability of predication.

## Figures and Tables

**Table 1:** Reference data of okra seed samples in calibration and validation sets.

	Calibration Set (*n* = 100)					External Validation Set (*n* = 20)				
	Min	Max	Mean	SD	SEL	Min	Max	Mean	SD	SEL
Isoquercitrin(%)	0.2923	0.5971	0.4612	0.080	0.0073	0.3193	0.5946	0.4624	0.073	0.0076
Quercetin-3-*O*-gentiobioside(%)	0.7150	1.733	1.249	0.26	0.027	0.7214	1.727	1.219	0.24	0.019
TP (%)	1.633	10.18	5.513	2.3	0.26	0.8219	9.700	5.482	2.1	0.23
DPPH (%)	0.4718	0.8936	0.7368	0.097	0.017	0.4327	0.8927	0.7358	0.086	0.0096
FRAP (mmol/g)	0.9214	11.72	4.119	2.3	0.24	1.072	10.64	4.158	2.4	0.27

TP: Total Polyphenols; DPPH: 1-diphenyl-2-picrylhydrazyl; FRAP: Ferric Reducing Antioxidant Power; SD: Standard Deviation; SEL: Standard Error (measurement of uncertainty) of Laboratory Method.

**Table 2:** Prediction results of antioxidants and antioxidant activity of okra seed samples by PLS with different reprocessing methods in calibration and prediction analysis.

Parameters	Preprocessing	Wavenumber Range (cm^−1^)	Calibration		Cross-Validation		
			$R_{C}^{2}$	RMSEC	$R_{CV}^{2}$	RMSECV	RPD
Isoquercitrin	FD + SNV	11,995.5~6098 4601.5~4246.1	0.9232	0.01760	0.9096	0.02600	4.07
	SNV	11,995.5~5446 4601~4246	0.9499	0.02260	0.9010	0.02400	3.18
	FD + SNV	11,995~7498 6101.9~5446.2 4601.5~4246.6	0.8155	0.02900	0.6216	0.03810	2.33
	MSC	11,995.5~6098 5450~4246.6	0.6953	0.03860	0.4368	0.04790	1.81
Quercetin-3-*O*-gentiobioside	FD + SNV	7502~5446.2; 4601.5~4246.6	0.9412	0.05890	0.9387	0.08690	4.04
	FD + MSC	7502~5446.2; 4601.5~4246.6	0.9318	0.06320	0.9281	0.08840	3.73
	FD + MSC	7502~4246.6	0.9293	0.06330	0.8197	0.09150	3.76
	MSC	7502~5446.2; 4601.5~4246.6	0.9208	0.06700	0.8172	0.09210	3.55
TP	FD + SNV	7502~5446.2; 4601.5~4246.6	0.9896	0.2620	0.9722	0.3870	9.79
	FD + COE	7502~5446.2; 4601.5~4246.6	0.9690	0.4600	0.8870	0.7430	3.08
	FD + MSC	7502~5446.2; 4601.5~4246.6	0.9406	0.607	0.8858	0.747	2.99
	MSC	7502~5446.2; 4601.5~4246.6	0.8809	0.7620	0.8555	0.9390	2.90
DPPH	MSC	11,995.5~4246.6	0.9798	0.01510	0.9522	0.02090	4.58
	SNV	11,995.5~7498; 5450~4246.5	0.9252	0.02750	0.8548	0.03640	2.63
	FD	11,995.5~4597	0.9746	0.01650	0.8541	0.03640	2.62
	COE	11,995.5~7498; 6101~4246.5	0.9212	0.02910	0.8406	0.03820	2.50
FRAP	MSC	7502~4246.5	0.9676	0.4730	0.9410	0.5700	4.12
	SNV	7502~5446.6	0.9715	0.4440	0.9401	0.5740	4.08
	FD	7502~4246.5	0.9724	0.4370	0.9400	0.5750	4.08
	MSC	7502~5446.6; 4601.5~4246.5	0.9686	0.4680	0.8443	0.9510	2.62

TP: Total Polyphenols; DPPH: 1-diphenyl-2-picrylhydrazyl; FRAP: Ferric Reducing Antioxidant Power; FD: First Derivative; SNV: Stand Normal Variate; MSC: Multiplicative Scatter Correction; SLS: straight line subtraction; COE: constant offset elimination; $R_{C}^{2}$: the coefficient of determination for calibration; $R_{\mathrm{CV}}^{2}$: the coefficient of determination for cross-validation; RMSEC: root mean square error of calibration; RMSECV: root mean square error of cross validation; RMSEP: root mean square error of prediction; RPD: residual predictive deviation.

**Table 3:** External validation of the established partial least squares (PLS) models for antioxidant constituents and activity.

Parameters	External Validation		
	$R_{p}^{2}$	RMSEP	RPD
isoquercitrin	0.9043	0.024	3.1
quercetin-3-*O*-gentiobioside	0.9423	0.050	4.7
TP	0.9732	0.34	6.4
DPPH	0.9775	0.0194	4.4
FRAP	0.9734	0.423	5.6

TP: Total Polyphenols; TF: Total Flavonoids; DPPH: 1-diphenyl-2-picrylhydrazyl; FRAP: Ferric Reducing Antioxidant Power.

## Supplementary Materials

The supplementary materials are available online.
