# Supplementary material for: Rapid Determination of Active Compounds and Antioxidant Activity of Okra Seeds Using Fourier Transform Near Infrared (FT-NIR) Spectroscopy
Source: Molecules. 2018 Mar 2;23(3):550. doi: 10.3390/molecules23030550 (PMC6017380; doi:10.3390/molecules23030550)
Supplement: Supplementary file 1 [file molecules-23-00550-s001.pdf]

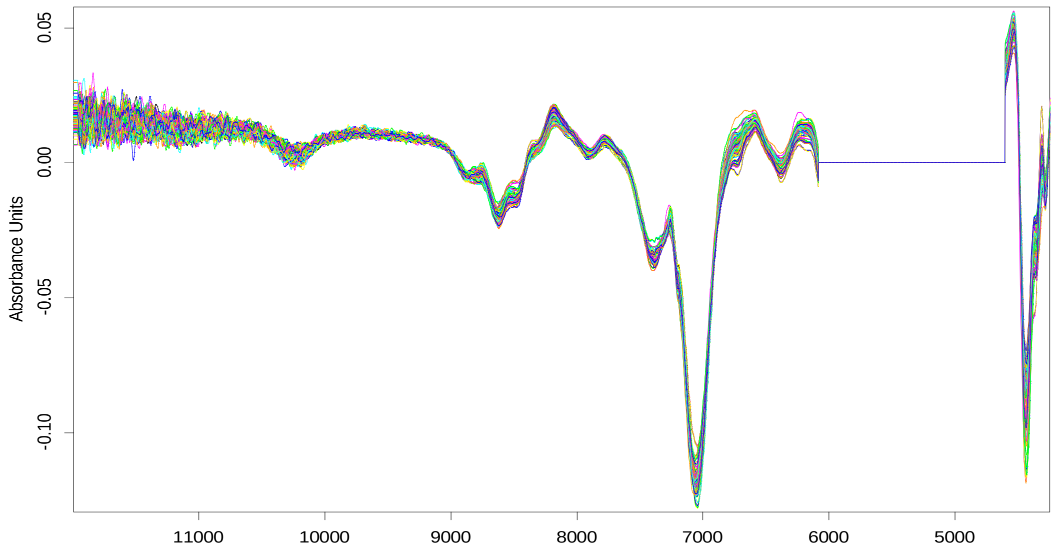

Figure S1. The NIR spectrum of okra seeds preprocessed with FD+SNV for isoquercitrin (11995.5~6098, 4601.5~4246.1 cm^{-1})

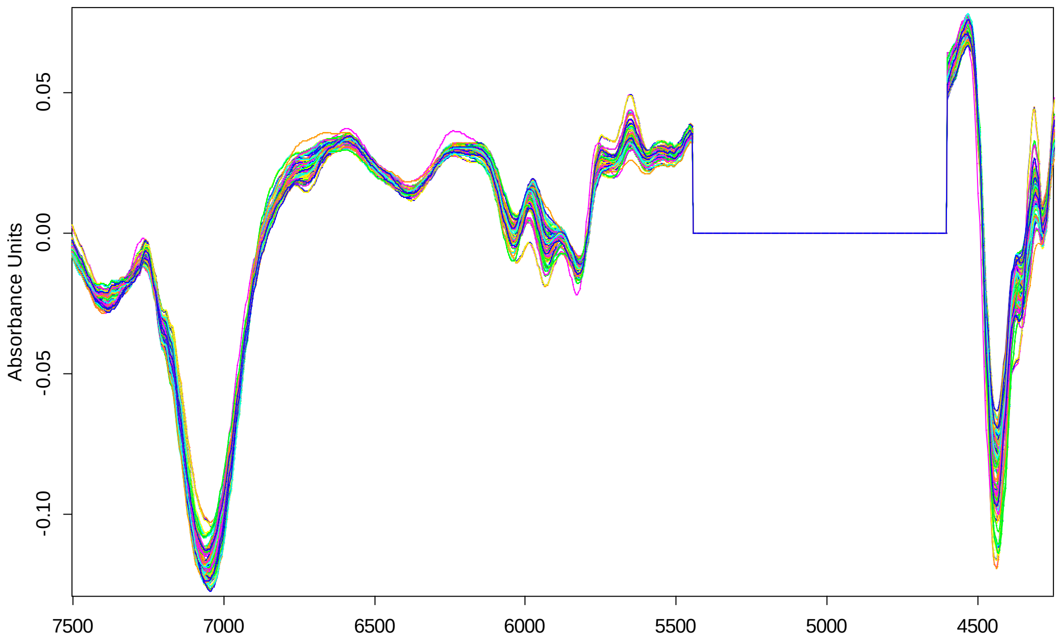

Figure S2. The NIR spectrum of okra seeds preprocessed with FD+SNV for quercetin-3-O-gentiobiose (7502~5446.2; 4601.5~4246.6 cm^{-1})

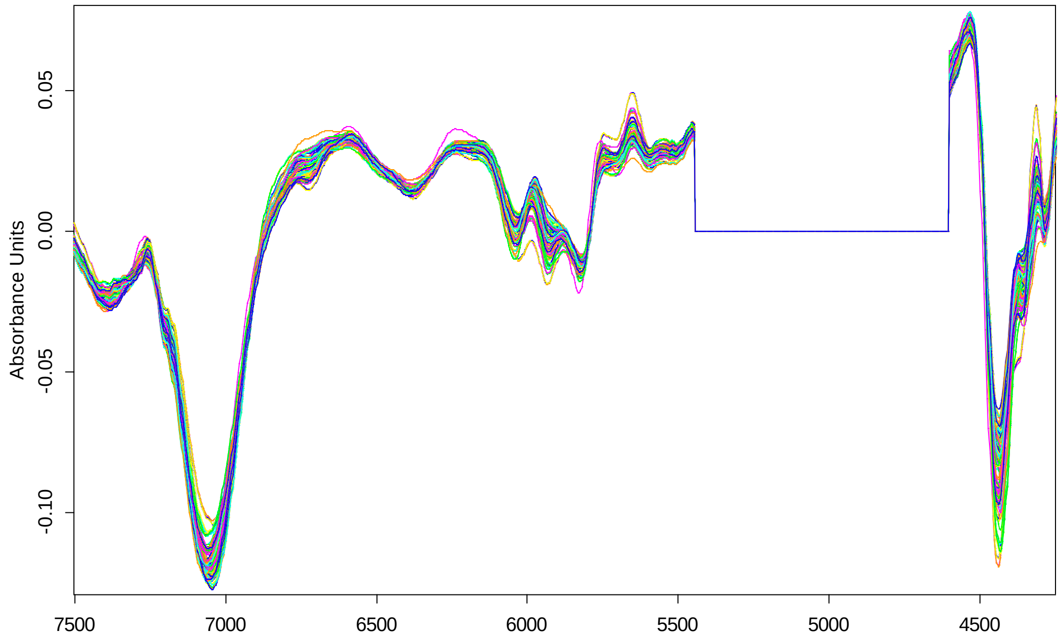

Figure S3. The NIR spectrum of okra seeds preprocessed with FD+SNV for total polyphenol (7502~5446.2; 4601.5~4246.6 cm^{-1})

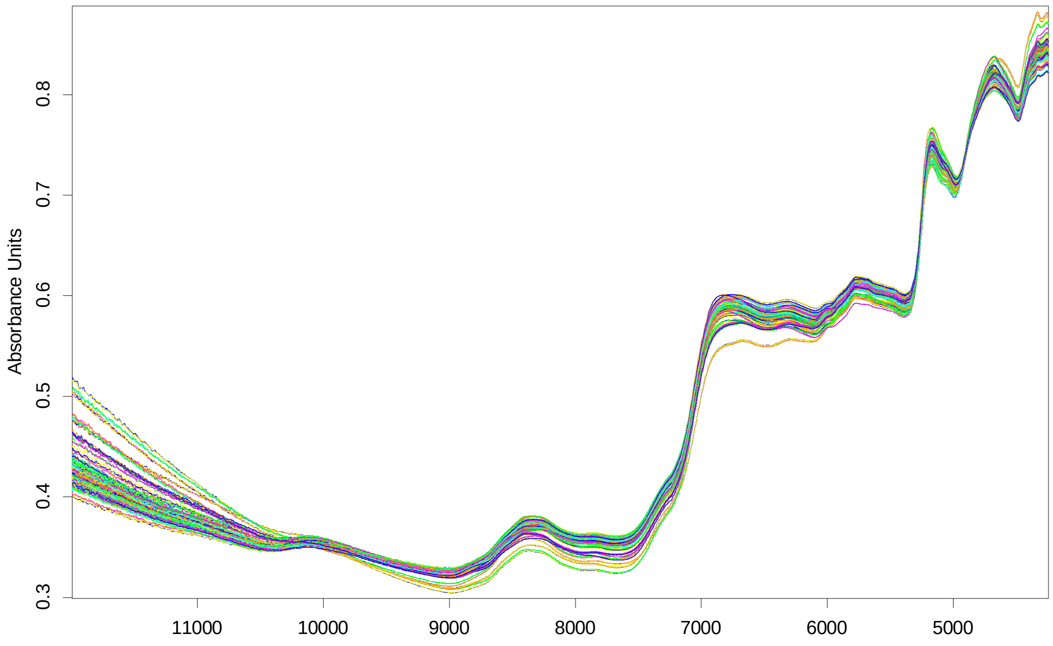

Figure S4. The NIR spectrum of okra seeds preprocessed with MSC for DPPH (11995.5~4246.6 cm^{-1})

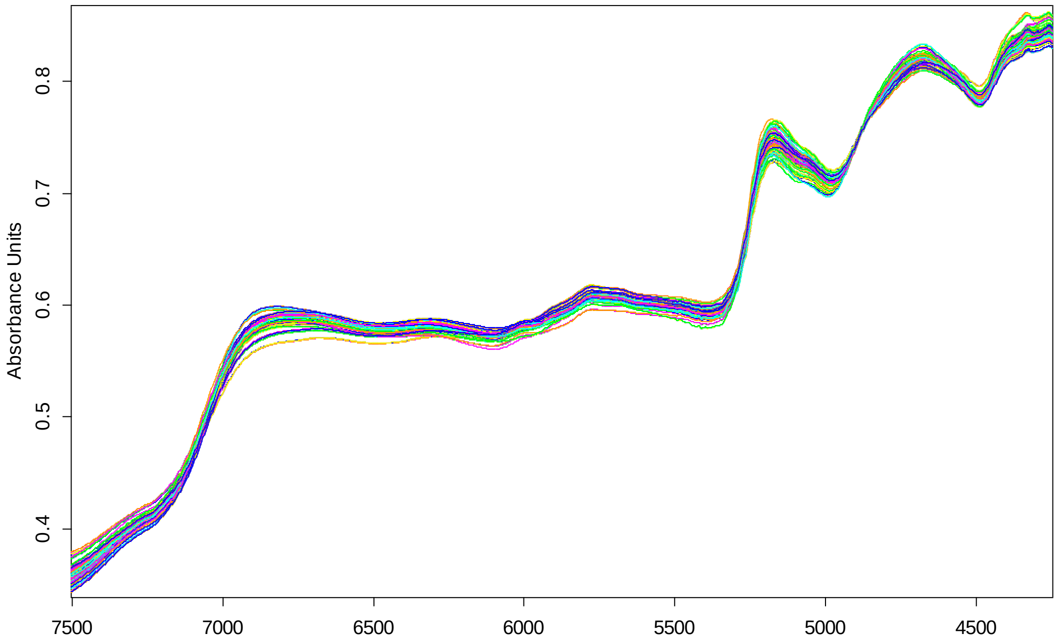

Figure S5. The NIR spectrum of okra seeds preprocessed with MSC for FRAP (7502~4246.5 cm^{-1})
